# CT and MR Studies of Giant Dermoid Cyst Associated to Fat Dissemination at the Cortical and Cisternal Cerebral Spaces

**DOI:** 10.1155/2013/239258

**Published:** 2013-01-16

**Authors:** Alessandro D'Amore, Alessandro Borderi, Rita Chiaramonte, Giorgio Conte, Ignazio Chiaramonte, Vincenzo Albanese

**Affiliations:** ^1^Department of Radiology, University Hospital of Catania, 95100 Catania, Italy; ^2^Department of Neurosurgery, University Hospital of Catania, 95100 Catania, Italy; ^3^Department of Otorhinolaryngology, University Hospital of Catania, 95100 Catania, Italy; ^4^Department of Neuroradiology, University Hospital of Catania, 95100 Catania, Italy

## Abstract

This study focuses on CT and MR studies of adult patient with giant lesion of the posterior cranial fossa associated with micro- and macroaccumulations with density and signal like “fat” at the level of the cortical and cisternal cerebral spaces. This condition is compatible with previous asymptomatic ruptured dermoid cyst. Histological findings confirm the hypothesis formulated using the imaging. We also integrate elements of differential diagnosis by another giant lesion of the posterior cranial fossa.

## 1. Introduction

Dermoid cyst is a congenital dysembryogenic lesion representing only 1% of intracranial lesions. The posterior cranial fossa is one of the possible development sites, and cystic growth is stimulated by many factors like sebum secretions and/or scaling of internal cystic wall.

Symptoms are related to dislocation and compression of brain parenchyma. The progressive lesion growth favours the rupture followed to cystic material dissemination in the cortical and cisternal brain spaces. In ruptured cyst the chemical meningitis can be subsequent. 

We present a case report of giant dermoid cyst on the posterior cranial fossa associated to micro- and macroaccumulation (also termed fatty droplets) widespread in the cortical and cisternal cerebral spaces. Elements of differential diagnosis from other giant lesion interesting the posterior cranial fossa have been integrated into the discussion.

## 2. Case Report

A 56-years-old woman was admitted at our neuroscience department complaining a recent slight diplopia and persistent headache which had arisen about five-six months before, initially NSAID responsive, that was progressively increasingly. A first medical examination showed peripheral right facial nerve and right abducent nerve paralysis. At the examination, vital signs and blood parameters were normal. No other neurological signs were detected.

**Figure 1:**
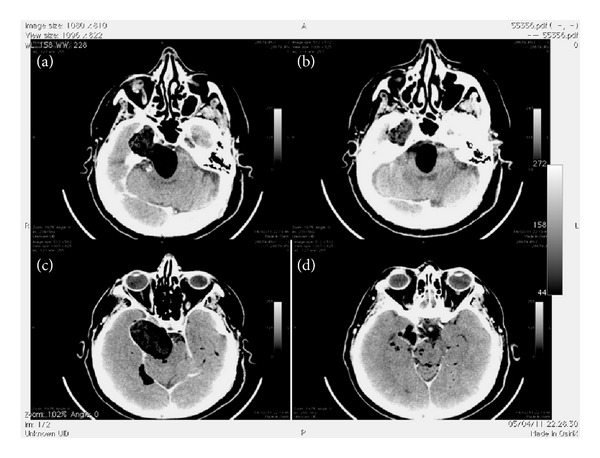
HRCT in axial plane showed heterogeneous hypodense lesion extending to middle and posterior fossa ((a) and (b)), and multiple bilateral hypodense collected disseminated along cerebral cisternal and cortical spaces ((c) and (d)).

A CT brain scan was performed with reconstruction in 2D MPR and VR that showed a presence of a giant expansive lesion on the right side of posterior cranial fossa (Figure 1(a)). 

The lesion developed from the posterior cranial fossa toward foramen magnum (Figure 1(b)) till middle fossa, causing a compression of right temporal lobe, that was displaced upwards and laterally, and a more serious compression of brainstem that was displaced on the left side. 

A cerebellar vermis on the IV ventricle was displaced too (Figure 1(a)) causing hydrocephalous.

Volume rendering and MPR images showed massive erosion of the right petrous apex and great wing of sphenoid, followed the diameter increasing of right inferior orbital foramen.

The densitometric difference evidenced during the examination showed an a cranial area iso-hypodense (−10/−5 HU) and a hypodense caudal area (−50/−60 HU) that seemed like a fat component. 

CT scan showed, moreover, a different marked hypodense lesions (−150/−180 HU) spreading over supratentorial cisternal and cortical spaces (Figures 1(c) and 1(d)). 

To reach a more comprehensive view about the lesion and its relationship with the intracranial circulation, an MR scan was performed.

The FSE T2-weighted, FLAIR, EPI, 3D T1-fat suppression, and 3D T1-enhancement sequences were performed on the three orthogonal planes. 

Moreover evaluation of intracranial circulation was assessed trough the acquisition of 3D-TOF, used for post-processing images like 3D-MIP and 2D-MPR.

**Figure 2:**
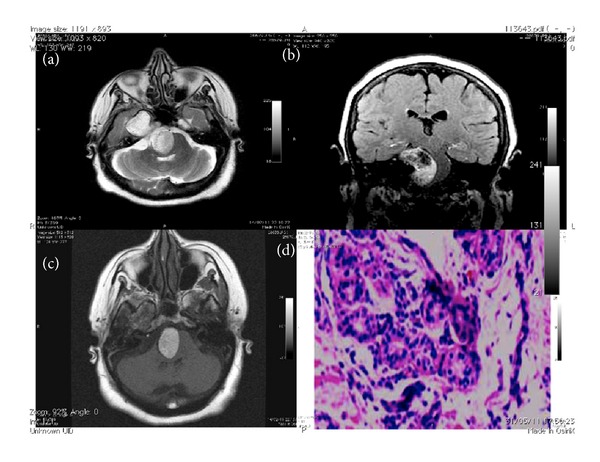
Axial FSE T2-w showed hyperintense lesion involving media and posterior fossa (a); coronal FLAIR showed a marked displaced brainstem by the lesion that resulting in the cranial portion disomogeneus hypo/hyperintense (b); axial SE T1-w showed marked hyperintense signal originating by caudal portion of the lesion (c); the histological sample obtained by biopsy showed recent and previous bleeding; hemosiderin pigments are well apparent (d).

The lesion caudal area appeared homogeneously hyperintense (Figures 2(a) and 2(b)) and heterogeneously hyper/hypointense signal in the cranial area both in SE T1-w, FSE T2-w and in FLAIR sequences.

The dark signal in the lesion was suggestive for hemosiderin presence linked by previous bleeding (Figures 2(b) and 2(c)). After intravenous administration of 12 mL of Gadolinium-DTPA, lesion didnot show contrast enhancement.

**Figure 3:**
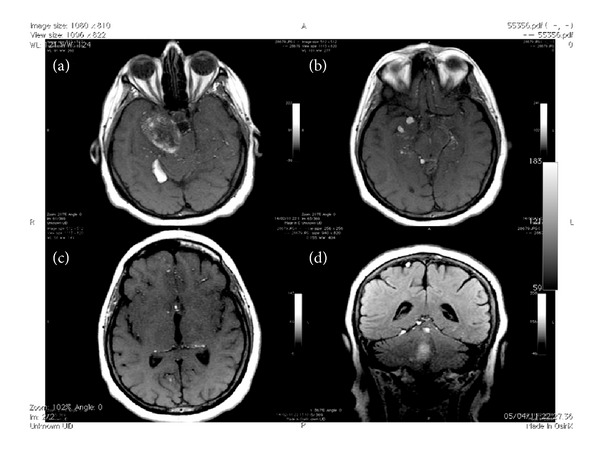
Axial SE T1-w images showed multiple bilateral micro- and macrolipid accumulations (fatty droplets ) into cerebral cisternal and cortical spaces ((a), (b), and (c)); coronal FLAIR showed hyperintense macrocollected interposed between occipital lobe and cerebellum and at the level of cerebral convexity (d).

The other multiple hyperintense areas in T2-w and T1-w images (linkable with fatty droplets) appeared spreading to the supratentorial cisternal and cortical spaces (from cerebral base to convexity); after contrast administration no enhancement was showed. (Figures 3(a), 3(b), 3(c), and 3(d)). 

In 3D-TOF sequences, we have found abnormal course of the basilar artery dislocated and stretched toward the left side, the right posterior cerebral artery that was raised and lengthened and the right posterior communicant artery that appeared extremely stretched.

The careful analysis of the acquired data provides the clue of the primary disorder: a giant dermoid cyst.

The patient underwent partial resection of the lesion with transtemporal approach whose aim was to reduce mass effect on the brainstem and right temporal bone.

During the surgery, many hair and some bone fragments escaped confirmed its origin.

By the way material was collected to perform the histological investigation which, at the last, confirmed the dysembryogenic nature of the lesion. (Figure 2(d)).

## 3. Discussion

Intracranial dermoid cyst is a congenital dysembryogenic lesion [1] that develops from inclusion of ectodermal elements during neural tube closure. This defect explains the propensity of dermoid cyst for the midline localization. 

Besides usually we do not find mesodermal elements and adipose tissue within this lesion.

Lunardi et al. originate intracranial dermoid cyst from ectodermic nest cells dysembryogenetically adherent to primitive veins [2].

It is a benign, ectopic, and squamous epithelial cyst containing dermal elements, including hair follicles, sebaceous, and sweat glands. This lesion accounts for 0,5%–1% of all intracranial lesion and is four to nine times less common than epidermoid cysts. Its development occurs between the 2nd and the 3rd decade of life and slightly prefers males. Sporadically, can be associated with Goldenhar (oculo-auriculo-vertebral dysplasia) and Klippel-Feil syndromes. When the entrapment of embryonic ectoderm tissue into the neural tube occurs between the 5th and 6th week of fetal life, the lesion usually will be localized in midline; if the entrapment occur later, during the formation of secondary cerebral vesicles, it will be easily ahead from the midline [1, 3]. The most frequent localization on the midline interesting sellar, suprasellar, and frontonasal regions. A less common localization is the posterior cranial fossa.

Our patient was presented with unilocular giant lesion starting from basal cisterns extending down towards the foramen magnum with great dislocation of the brainstem and the cerebellar vermis. 

In the cranial site, it was pushing the tentorium brain invading the right middle cranial fossa and causing compression of right temporal lobe. 

The pathophysiology of these is related to their progressive growth with consequent compression and dislocation of nearby neurovascular structures. 

Its growth is supported by endocrine influence that is increasing with age, in that case the dermoid cysts can secrete sebum and sweat, and, sometimes, exfoliating squamous epithelium can be found in the cyst. By that, this injury is often revealed only in adulthood, and its progressive growth is responsible of rupture and subsequent, possible, chemical meningitis.

The most common symptoms are headache (32%) and seizure (30%) and when the cyst becomes particularly large, symptoms will be related to compression of neurovascular structures and eventually developing of cerebral hypertension syndrome. 

Chemical meningitis occurs in a high percentage of patients after rupture of dermoid cyst. The possibility of malignant degeneration in the sense of squamous cell carcinoma is rare but possible complication [2].

On CT images, this lesion appears like a uniloculated, well-delineated, cystic mass with hypodensity from moderate to significant (from 0 to −150 HU), sometimes are present capsular calcifications. Generally, does not show enhancement after contrast administration.

MR findings [3, 4] are hyperintense signal on SE T1 sequences and heterogeneous hypo-hyperintense signal on FSE T2. Typically, it is possible to be recognized on FSE T2-w sequence hypointense lines inside the lesion corresponding to hair contained in the cyst. Fat-suppression sequence can confirm the lipid presence into the lesion. The 3D chemical shift selective sequences can be used to increase diagnostic specificity [3]. After contrast administration, minimal enhancement of capsule was shown without central enhancement; the lipid subarachnoid accumulations do not show enhancement; in a patient with ruptured cyst, the presence of extensive leptomeningeal enhancement is indicative of the development of chemical meningitis.

The “fat” density or signal seen in the images is not due to the presence of adipose tissue but depends on the internal composition of the lesion like cholesterol. Heterogeneous aspect depends on a keratinaceous debris, sebaceous and sweat secretion as well as appendages including hair, and rarely nails or teeth.

In our case it has been possible to detect the presence of multiple micro- and macroaccumulation in the cisternal and cortical cerebral spaces: this finding is typically compatible with previous asymptomatic rupture. 

The diffusion which is also linked to progressive growth of the lesion allowed to release cholesterol crystals in the subarachnoid spaces which resulting in widespread of lipid collected. This condition is frequently reported and well documented after rupture of intracranial dermoid and epidermoid cysts [5–7]. Rupture may occur spontaneously or resulting from trauma or surgery event [8].

When the cholesterol crystal is spread by cerebral spinal fluid (CSF) it tends to migrate into subarachnoid spaces particularly at the level of cerebellopontine angle, suprasellar and prepontine cistern and along the sagittal sinus. 

In our patient, massive dissemination was represented by macro and microcollect of fatty elements.

Lipoid aseptic meningitis [9] is the most common complication of fat dissemination linked with meningeal irritation caused by cholesterol crystal; the only presence of fat in the CSF spaces is not automatically associated with this particular form of meningitis (like in the case of our patient).

Histopathological result of the biopsy confirmed the neuroradiological suspect.

In our case, erosion of the petrous apex and sphenoidal great wing with an increasing diameter of the inferior orbital foramen made opportunely a differential diagnosis with giant cholesteatoma and cholesterol granuloma [10]. The chance of an injury starting from the petrous apex was discarded because the 2D and 3D reconstructions showed symmetry of petrous apex between right and left sides; a bone erosion found in right petrous apex was coming from external. For that, cholesteatoma, or particularly cholesterol granuloma (the most frequent giant lesion of the petrous apex), was excluded from the differential diagnosis. We trust that CT scan and postprocessing reconstruction in MIP e VR are essential for the exact localization of lesion origin.

The most important differential diagnosis from other intracranial midline lesions includes epidermoid cyst, craniopharyngioma, teratoma, and lipoma.

*Epidermoid cyst* (called cholesteatoma if localized in the middle ear) arises from normal epithelial cells included during neural tube closure; this lesion tends to become “giant” through a progressive accumulation of cholesterol and keratin due to the desquamation of the lining epithelium, like dermoid cyst. The imaging difference between them on CT scan is that epidermoid cyst presents hypodense with HU score similar to the CSF; also bone erosion is present. On MR scan shows often slightly hyperintense to CSF on T1-w images; less commonly is hyperintense to brain due to high contents of triglycerides and unsaturated fatty acids on T1-w (also termed “white epidermoid”). When solid crystal cholesterol and keratin predominate the lesion appears hypointense to CSF on T1-w (also termed “black epidermoid”). On T2-w images, it is often isointense or slightly hyperintense to CSF. On DWI, it is characteristic hyperintense; often, it is necessary to differentiate them from arachnoid cyst [4], and it become possible by the analysis of the apparent diffusion coefficient (ADC) on EPI sequence: epidermoid cysts have a similar ADC to brain parenchyma, while arachnoid cysts have ADC similar to free water. After contrast administration does not show enhancement, enhancement lesion is a sign of malignant degeneration.

The distinction between dermoid and epidermoid cysts is prognostically relevant and may impact on surgical management. In dermoid, the surgical choice is usually represented by subtotal resection also because it is rarely followed by lesion recurrence. In epidermoid, it prefers a total resection. Furthermore, the epidermoid cystic has a low incidence of rupture also because its growth is not regulated by hormonal factors but only by squamous exfoliation of inning layer.

The difference from the *teratoma*, as well as being possible through the “Imaging,” is that it take origin from all three germinal layers (ectoderm, mesoderm, and endoderm) [1, 4], instead dermoid cysts have tissue represented only by ectodermal layer. Unlike the dermoid cysts it does not present fat fluid level and often shows multicystic aspect or multiloculated organization. By the way, those of the the CT and MR characteristics of the lesion content cannot be easily distinguished from dermoid cysts.

Teratoma has also extremely heterogeneous signal: iso-hyperintense both T1-w and T2-w due to the presence of calcification, CSF, fat, and soft tissue components; typically, teratoma may contain adipocytes (to the opposite of dermoid). On DWI, the solid component which shows a restricted diffusion after contrast administration enhancement of the soft tissue is typical.

Intracranial *lipoma* is a well-delineated mass of mature nonneoplastic adipose tissue often associated with agenesis of the corpus callosum; it is usually more homogeneous than dermoid cyst and capsular calcification has rarely. Lipoma shows marked hypodense on CT scan (−50 to −100 HU); on MR scan, it is hyperintense on T1-w and hypointense on T2-w and fat suppression, without contrast enhancement.

*Craniopharyngioma* is an aggressive lesion often found in midline including the intra- and suprasellar region with serious skull-based erosion, frequently finding on CT images. In childhoods (adamantinomatous type), usually presents a solid (isodense) mass whit cystic (hypodense) component (sometimes multi cystic); nodular calcifications and nodular or rim enhancement are frequent findings. In adults (papillary type), lesion shows isodense with nodular enhancement.

Common MR findings for the solid component are heterogeneous signal on T1-w and T2-w images with heterogeneous enhancement; cystic components are variably hyperintense on T2-w while signal varies with the cyst contents on DWI and T1-w images (short T1 due to high protein content), after contrast cyst wall shows strongly enhancement. Dermoid cyst showed minimal or no enhancement. This lesion is more common than dermoid cyst, and normally it is easily to differentiate from other intracranial lesions in the midline. 

CT and MR images have been relevant for correct diagnosis that allows the most appropriate choosing for a better surgical procedure case by case. 

The most frequently selected surgical approaches are the middle cranial fossa or, if possible, transtemporal that provides the widest surgical view preserving acuity of hearing. 

Our patient has been undertaken to transtemporal approach with complete excision of the lesion, but, in absence of previous medical history of meningitis, it was not considered necessary to remove the lipid macrocollected.

Total removal is not recommended when these lesions adhere to the neurovascular structures as this is associated with high morbidity and mortality; in our case, the lesion showed a surgical cleavage plane without adhesions with the neurovascular structures of the middle and posterior fossa. 

The possibility of recurrence should, therefore, be considered especially when the capsule is not completely removed. 

Two weeks after intervention, our patient showed complete remission of the symptoms.

Subsequent CT and MRI followup showed normal postsurgery course.
